# Acute Myocardial Infarction in a Hospital Cohort of Malaria

**DOI:** 10.4103/0974-777X.59258

**Published:** 2010

**Authors:** Karun Jain, M Chakrapani

**Affiliations:** *Department of Orthopedics, JSSMC, Mysore - 570004, India*; *Department of Medicine, KMC, Mangalore (Manipal University) - 575001, India*

Sir,

Malaria, a protozoal disease, caused by genus plasmodium, is prevalent in about 100 countries worldwide[1] and is a major cause of morbidity and mortality especially in sub-Saharan Africa, Southeast Asia, and Latin-America.[2] In India about 1.65 million cases were reported (with 943 deaths) during the years 2003 and 2004.[1] Malaria is an endemic disease in the city of Mangalore, Karnataka, since 1994-1995.

Cardiac involvement in malaria has not been studied widely. There have been few reports of experimental and postmortem studies indicating myocardial involvement in malaria.[2–7] We investigated the extent of cardiac involvement in malaria in the clinical situation, by analyzing the occurrence of acute myocardial infarction (AMI) in patients with malaria and comparing it with AMI in nonmalarial patients.

A retrospective observational study of 38,919 in-patients of Dr. TMA Pai Rotary Hospital, Mangalore, was done from the year 1995 to 1998, and it was found that among 1531 malarial patients, 22 had AMI (1.43%), a statistically significant (*P* < 0.05) occurrence, as compared to AMI among all in-patients who were in for complaints other than malaria, (0.82%), reflecting the possibility of myocardial damage in malaria. Analysis had been started from 1995, as malaria resurged in Mangalore city from 1995 onwards. Diagnosis of malaria cases had been established by the Quantitative Buffy Coat (QBC) test[8] and diagnosis of myocardial infarction had been established by the treating physicians following standard electrocardiogram (ECG) changes and cardiac biomarker profiles.

The occurrence of AMI was higher among in-patients with malaria compared to in-patients without malaria from 1995 to 1998. [Tables 1 and 2]. Out of 22 cases of AMI among patients with malaria, 13 patients had *P. falciparum* malaria, two patients had *P. vivax* malaria, and seven patients had mixed malaria (*P. falciparum* + *P. vivax*).

**Table 1:** Year-wise and cumulative analysis of occurrence of acute myocardial infarction among patients with malaria and acute myocardial infarction among all other in-patients for four years

Year	AMI among malaria inpatients (n = 22) (%)	AMI among all inpatients (other than due to malaria) (n = 309) (%)	*P* value
1995	9/365 (2.47)	96/11005 (0.87)	< 0.001
1996	6/465 (1.29)	97/11113 (0.87)	< 0.001
1997	4/418 (0.96)	75/8646 (0.87)	Not significant
1998	3/238 (1.06)	47/6624 (0.71)	Not significant
Total	22/1531 (1.43)	309/37388 (0.82)	< 0.05

AMI: Acute myocardial infarction

**Table 2:** Demographic profile of acute myocardial infarction among patients with malaria and acute myocardial infarction among all other in-patients

	AMI among malaria in-patients (n = 22)	AMI among all in-patients (other than due to malaria) (n = 309)
Age (years)	53.05 ± 5	56.20 ± 5
Sex (M:F)	3.67:1	4.43:1

AMI: Acute myocardial infarction

The pathophysiological link between myocardial damage and malaria has been described in literature.[3,6,7,9] Adhesion of parasitized red blood cells to the endothelium of myocardial capillaries has been shown in monkeys and man.[3,9] Ischemia, acidosis, toxic effects of substances similar to *P. falciparum* glycosyl-phosphatidyl-inositol or Plasmodium-triggered mechanisms such as apoptosis may be responsible for myocardial damage.[6] Raised catecholamine has been found in malaria, which may induce vasoconstriction, resulting in myocardial damage.[3]

An interesting observation was the gradual reduction in occurrence of AMI as the years progressed. While the occurrence of AMI among all in-patients without malaria remained stable at about 0.8% over the study period, the occurrence of AMI among patients with malaria decreased from 2.4% in 1995, when there was a resurgence of malaria in Mangalore, to 1.1% in 1998. This might be because of the gradual development of immunity in this area as the population was continuously exposed to the malarial parasite. In hyper endemic malarial areas patients could tolerate high parasite density, even up to 20-30%, often without clinical symptoms, advocating looking for cardiac complications in non-immune individuals suffering from malaria.

Although this observation does not imply a cause-effect relationship, temporal changes over the four years and a possible biological explanation from the previous studies[3,6,7] suggest that malaria could have been the cause of the higher occurrence of AMI in this group. We provide the first study in a hospital setting, demonstrating the cardiac complications, that is, acute myocardial infarction, in malaria.

Further prospective research could provide more details. In conclusion, we propose that AMI should be regarded as an important clinical complication of malaria. This is of importance, as it is known that some of the anti-malarial drugs also depress cardiovascular function.
